# Supplementary material for: Evolutionary pathways of repeat protein topology in bacterial outer membrane proteins
Source: eLife. 2018 Nov 29;7:e40308. doi: 10.7554/eLife.40308 (PMC6340704; doi:10.7554/eLife.40308)
Supplement: Figure 2—source data 1. — Average E-values calculated in log space of the prototypical barrels, within and between barrels of different sizes. Only interactions with E-values <10–3 were included in the average. The minimum value of each row has been bolded. Values shown are the average E-values calculated in log space. [file elife-40308-fig2-data1.pdf]

| Average E-value, SeqID <= 25% | | | | | | | |
|---|---|---|---|---|---|---|---|
| Strands per Chain | 8 | 10 | 12 | 14 | 16 | 18 | 22 |
| 8 | **6.40e-15** | 4.43e-05 | 1.69e-06 | 1.83e-06 | 3.11e-05 | | |
| 10 | NA | **1.10e-14** | 1.41e-04 | 9.40e-04 | NA | NA | NA |
| 12 | | | **4.40e-27** | 1.39e-04 | 7.49e-06 | | 6.16e-04 |
| 14 | NA | NA | NA | **1.61e-35** | 6.89e-11 | NA | 1.23e-04 |
| 16 | | | | | **6.53e-37** | 2.29e-05 | |
| 18 | NA | NA | NA | NA | NA | **6.12e-32** | NA |
| 22 | | | | | | | **3.13e-52** |
