# Supplementary material for: Evolutionary pathways of repeat protein topology in bacterial outer membrane proteins
Source: eLife. 2018 Nov 29;7:e40308. doi: 10.7554/eLife.40308 (PMC6340704; doi:10.7554/eLife.40308)
Supplement: Figure 4—source data 1. — Longest sequence alignments between barrels of different sizes at E-values ≤ 10–3. The colors - red, orange, yellow, green, cyan, blue, purple, plum - represent strand numbers 1,2,3,4,5,6,7,8 in that order. For example, in the second alignment, red is aligning to cyan signifying that strand one aligns with strand 5. If higher number strands align then the colors wrap around again so that red to plum represent 9–16, etc. In all alignments, the smaller barrel is the top sequence; the larger barrel is the bottom sequence. [file elife-40308-fig4-data1.pdf]

8- to 10-stranded Longest Alignment, IntNum 1356

```
3qra A      2  EGESSISIGYAQSRVKEDGYKLDKNPR...GFNLKYRYEFNND..WGVIGSFAQTRRGFE     56
2vdf A      7  ANEFTVHTDLSSISSTRAF..LKEKHKAAKHIGVRADIPFDANQGIRLEAGFGRSKKNII    64

3qra A     57  ESVDG.........FK.LIDGDFKYYSVTAGPVFRI..NE..YVSLYGLLGAGHGKAKFS    102
2vdf A     65  NLETDENKLGKTKNVKLPTGVPENRIDLYTGYTYTQTLSDSLNFRVGAGLGFESSKDSIK    124

3qra A    103  S.IFGQSESRSKTSLAYGAGLQFNPHPNFVIDASYEY   138
2vdf A    125  TTKHTLHSSRQSWLAKVHADLLSQLGNGWYINPWSEV   161
```

8- to 12-stranded Longest Alignment, IntNum 703

```
2maf A      8  GPYVQADLAYAYEHITHDYPEPTAPNKNKISTVSDYFRNIRTRSVHPRVSVGYDFG...G    64
1uyn X    120  IGYLKGLFSYGRYKNSISRSTGA.........DEHAEGSVNGTLMQLGALGGVNVPFAAT   170

2maf A     65  WRIAADYA.RYRKWNNNKYSVNIENVRIRKENGIRIDRKTENQE.NGTFHA.VSSLGLSA   121
1uyn X    171  GDLTVEGGLRYDLLKQDAFAEKGSAL.............GWSGNSLTEGTLVGLAGLKL   216

2maf A    122  IYDFQINDKFKPYIGARVAYGHVRHSIDSTKKTIEVTTVPSNAPNGAVTTYNTDPKTQND   181
1uyn X    217  SQPLS..DKAVLFATAGVERDLNGRDYTVTGG...F...............TGATAATGK   256

2maf A    182  YQSNSIRRVGLGVIAGVGFDITPKLTLDAGYRYHNWGRLENTRFKTHEASLGVRYRF   238
1uyn X    257  TGARNMPHTRLVAGLGADVEFGNGWNGLARYSYA.GSK....QYGNHSGRVGVGYRF   308
```

8- to 14-stranded Longest Alignment, IntNum 1494

```
2x27 X      4  GHKAGDFIIRGGFATVDPDDSSSDIKLDGAKQRGTKATVDSDTQLGLTFTY..MFAD.KW    60
4d5b A    162  NDYNMGFYSNLEYLLSKEDRNAW..............GKRQEQGYSALFKPYKRFGNWEV   207

2x27 X     61  GVELVAATPFNHQVDVKGLGPGLDGKLADIKQLPPTLLLQYYPMGGTNSAFQPYGGLGVN   120
4d5b A    208  GVEFYYQIKT.NDEKQPDG...TINEKSDFNERYIEPIVQYSFD....DAGTLYTRVRVG   259

2x27 X    121  YTTFFDEDLASNRKAQGFSSMKLQDSWGLA.....GELGFDYMLNEHALFNMAVWYMDID   175
4d5b A    260  KNETKNTDRS..........GGGNAGINYFKDIRKATVGYEQSIGESWVAKAEYEYANEV   309

2x27 X    176  TKAS.INGPSALGVNKTKVDVDVDPWVYMIGFGYKF   210
4d5b A    310  EKKSRLSG......WEARNKSELTQHTFYAQALYRF   339
```

8- to 16-stranded Longest Alignment, IntNum 529

```
1qj8 A      3  STVTGGYAQSDAQG........QMNKMGGFNLKYRYEEDNSPLGVIGSFTYTEKSRTASS    54
5fvn A    188  FGIGTAVSSSKRTSSQNDLTYGNGDRAETYTGGLKYDANN..IYLAAQYTQTYNATRVG.   244
```

```
1qj8 A    55  GDYNKNQYYGITAGPAYRINDWASIYGVVGVGYGKFQTTEYPTYKNDTSDYGFSYGAGLQ  114
5fvn A   245  NLGWANKAQNFEVVAQYQFDFGLRPSVA..YLQSKGKDLEN...GYGDQDLLKYVDVGAT  299

1qj8 A   115  FNPMENVALDFSYEQSRIRS.........VDVGTWIAGVGYRF   148
5fvn A   300  YYFNKNMSTYVDYKINLLDDKEFTRNAGISTDDIVALGLVYQF   342
```

10- to 12-stranded Longest Alignment, IntNum 1348

```
2vdf A    30  HKAAKHIGVRADIPFDANQGIRLEAGFGRSKKNIINLETDENKLGKTKNVKLPTGVPENR   89
3aeh A   105  KTKSWGGGFYASGLFRSGAYFDVIAKYIHNENKYDLNFA..........GAGKQNFRSHS  154

2vdf A    90  ..IDLYTGYTYTQTLSDSLNFRVGAGLGFESSKDSIKTTK...HTLHS..SR.QSWLAKV  141
3aeh A   155  LYAGAEVGYRYH..LTDTTFVEPQAELVWGRLQGQTFNWNDSGMDVSMRRNSVNPLVGRT  212

2vdf A   142  HADLLSQLGN.GWYINPWSEVKFDLNSRYKLNTGVTNLKKDI.NQKTNGWGFGLGANIGK  199
3aeh A   213  GVVSGKTFSGKDWSLTARAGLHYEFDLTDSADVHLKDAAGEHQINGRKDSRMLYGVGLNA  272

2vdf A   200  KLGESASIEAGPFYK   214
3aeh A   273  RFGDNTRLGLEVERS   287
```

12- to 14-stranded Longest Alignment, IntNum 712

```
1uyn X    39  NGTGLRVIAQTQQDGGTWEQGGVEGKMRGSTQTVGIAAKT..GENTTAAATLGMGRSTWS   96
2x9k A    39  ANGPWRIALAYYQEGP.VDYSAGKRGTWFDRPELEVHYQFLENDDFSFGLTGGFRNYGYH   97

1uyn X    97  EN..SA..NAKTDSISLFAGIRHDAGDIGYLKGLFSYGRYKNSISRSTGADEHAEGSVNG  152
2x9k A    98  YVDEPGKDTANMQRWKIAPDWDVKLTDDLRFNGWLSMYKFANDLN.........TTGYAD  148

1uyn X   153  TLMQLGALGGVNVPFAATGDLTVEGGLRYDLLKQDAFAEKGSALGWSGNSLTEGTLVGLA  212
2x9k A   149  TR..VETETGLQYTFNETVALRVNYYLER......GFNMDD.....SRNNGEFSTQEIRA  195

1uyn X   213  GLKLSQPLSDKAVLFATAGVER.DLNGRDYTVTGGFTGATAATGKTGARNMPHTRLVAGL  271
2x9k A   196  YLPLTLG.NHSVTPYTRIGLDRWSNWDWQ.......D.........DIEREGADFNRVGL  238

1uyn X   272  GADVEFGNGWNGLARYSYAGSKQ.....YGNHSGRVGVGYRF   308
2x9k A   239  FYGYDFQNGLSVSLEYAFEWQDADEGDSDKFHYAGVGVNYSF   280
```

12- to 16-stranded Longest Alignment, IntNum 1395

```
2wjr A    27  NGWWASMESNTWNTIHDNKKENAALNDVQVEVNYAIKLDDQWTVRPGMLTHFSS......   80
3nsg A   147  DGLSFGIQYQGKNQDNHS.INSQNGDGVGYTMAYEF...DGFGVTAAYSNSKRTNDQQDR  202

2wjr A    81  ...NGTRYGPYVKLSWDATKDLNFGIRYRYDWKAYRQQDL..SGDMSRDNVHRWDGYVTY  135
3nsg A   203  DGNGDRAESRAVGAKYDAN.NVYLAAVYAETRNMSIVENTVTDTVEMANKTQNLEVVAQY  261
```

```
2wjr A   136   HINSDFTFAWQTTLYSKQNDYRYANHKKWATENAFVLQYHMTPDITPYIEYDYLDRQGVY   195
3nsg A   262   QFDFGLRPAISYVQSKGKQLNGAGGSADLAKYIQAGATYYFNKNMNVWVDYRFNLLDEND   321

2wjr A   196   N.GRDNLSENSYRIGVSFKL   214
3nsg A   322   YSSSYVGTDDQAAVGITYQF   341
```

14- to 22-stranded Longest Alignment, IntNum 1859

```
3bry A   134   LVLRVPFSAAYHVTDKLTVGASVDAVWTSLNLGTLLDVSQIGTLAGQGRVSGTLVPTLLG   193
5fok A   424   KTSAAYVFDTLKLSEQWELNLGLRYDDFDTKSSGYQTAGRN...................   464

3bry A   194   VPGLSGGYIDFSRNAPVGGGVQAWGIGGRLGLTYQVTPDTRIGAAYQAKTHVGDLTGGQAT   253
5fok A   465   GP...AGYFK.........RENNSHFWNYQTGLVYKPAPNGSIYLAWSTSSNPTGETG...   510

3bry A   254   LSAVSSVAGNIPLKGDVTVRNFQMPAQLTVGISHQFN.DQLSVSADYQRVFWSSVMKDMN   312
5fok A   511   GEGQAD....I.SVGNN.GLDPERNRNLELGTKWAFFDDALSLNAALFRTDKTNARVASP   564

3bry A   313   VGFVQSGSAANLDLSLPQNYRDISVFGIGAEYRYNAKWTFRGGFHYAQEAIPGNM....L   368
5fok A   565   ...DV......STLQVLDGEQRVQGVELGFNGKLTEKWKVFGGYTYLDSEIRKSTVKSDE   615

3bry A   369   LAVVPATPTTSLTGGVSYAIGKNDVIDFALSVALR   403
5fok A   616   GNKMPQTAQNNFTLWTTYDLLQNFTIGGGTTYVDK   650
```

16- to 18-stranded Longest Alignment, excluding Fig 6, IntNum 2149

```
3nsg A     9   NKLDLYGKAVGRHVWTTTGDS....KNADQTYAQIGFKGETQINTDLTGFGQWEYRT...    61
3sys A     7   EDAKTDLVLRNYYFNRDFRDHDAGKSLVDEWAQGFILKFSSGYTPG.TVGVGLDAIGLFG    65

3nsg A    62   .KADR.....AE........GEQQNSNL.VRLAFAGLKYAEVGSIDYGRNYGIVYDVESY   106
3sys A    66   VKLNSGRGTSNSELLPLHDDGRAADNYGRVGVA.AKLRV.SASELKIGEMLPDIPLLR.Y   122

3nsg A   107   TDM..APY.FSGETWGGAYTDNYMTSRAGGLLTYRNSDFFGLVDGLSFGIQYQGKNQDNH   163
3sys A   123   DDGRLLPQTFRGF................AVVSRE..L....PGLALQAGRFDAVSLRN   159

3nsg A   164   SI...........NSQNGDGVGYTMAYEFD..GFGVTAAYSNSKRTNDQQDRDGNGDRAE   210
3sys A   160   SADMQDLSAWSAPTQKSDGFNYAGAEYRFNRERTQLGLWHGQLED...........VYR   207

3nsg A   211   SRAVGAKYDA..N..NVYLAAVYAETRNMSIVENTVTDTVEMANKTQNLEVVAQYQFDFG   266
3sys A   208   QSYANLLHKQRVGDWTLGANLGLFVDRDDGAAR.......AGEIDSHTVYGLFSAGIG.L   259

3nsg A   267   LRPAISYVQSKGKQLNG........A........GGSADLAKYIQAGATYYFN....KNM   306
3sys A   260   HTFYLGLQKVGGDSGWQSVYGSSGRSMGNDMFNGNFTNADERSWQVRYDYDFVGLGWPGL   319

3nsg A   307   NVWVDYRFNLLDENDYSSSYVGTDDQAAVGITYQ   340
3sys A   320   IGMVRYGHGSNATTKA..GSGGKEWERDVELGYT   351
```

14- to 16-stranded Longest Alignment, IntNum 1879

```
3bs0 A    92   DWRFGAGLFVSSGLGTEYGSKSFLSQTENGIQTSFDNSSRL.IVLRAPIGFSYQATSKLT   150
4y25 A    57   QFSEGKGIVRDWLAGVEWRSRNIWLEA..EYA...ERVFNHEHKPGARLSGWYDFNDNWR   111

3bs0 A   151   FGASVDLVWTSLNLELLLPSSQVGALTAQGNLSGGLVPSLAGFVGTGGAAHFSLSRNSTA   210
4y25 A   112   IGSQLERLSHRVPLRA............................................   127

3bs0 A   211   GGAVDAVGWGGRLGLTYKLTDNTVLGAMYNFKTSVGDLEGKATLSAISGDG.A.V..LP.   265
4y25 A   128   .MKNGVTGNSAQAYVRWYQNERRKYGVSWAF.T...DFSDSNQRHEVSLEGQERIWSSPY   182

3bs0 A   266   LDGDIRVKNFEM..PASLTLGLAHQFNE..RWVVAADIKRAYWGDVMDSMNVAFISQLGG   321
4y25 A   183   LIVDFL.PSLYYEQNTEHD.TPYYNPIKTFDIVPAFEASHLLWRSYENSWEQIFSAG.VG   239

3bs0 A   322   IDVALPHRYQDITVASIGTAYKYNNDLTLRAGYSYAQQALDSELILPVIPAYLKRHVTFG   381
4y25 A   240   ASWQKHYGTDVVTQLGYGQRISWNDVIDAGATLRWEKRPYDGDR.......EHNLYVEFD   292

3bs0 A   382   GEYDFD   387
4y25 A   293   MTFRFR   298
```
