# Supplementary material for: Evolutionary pathways of repeat protein topology in bacterial outer membrane proteins
Source: eLife. 2018 Nov 29;7:e40308. doi: 10.7554/eLife.40308 (PMC6340704; doi:10.7554/eLife.40308)
Supplement: Figure 5—source data 1. — Full alignments for diagrams in Figure 5. Strands are colored - red, yellow, green, cyan, blue, lavender – to represent the strand numbers 1–6. In all alignments, the 16-stranded barrel is the top sequence and the 18-stranded barrel is the bottom sequence. [file elife-40308-fig5-data1.pdf]

Large Rearrangements

Int Num 317
E-value 1.1E-6

```
1pho_A 007   DGNKLDVYGKVKAMHYMSDNA-S--------KDGDQSYIRFGFKGETQINDQLTGYGRWEAEFAGNKA-----ESDTAQQKTRLAFAGLKYK-DLGSFDY   092
51dv_A 009   AIKDVDVSGVLRYRYDTGNFDKNFLNNSNLNNSKQDHKYRAQVNFSAAIADNFKAFVQFDYNAVDGGTGVDNATNAEKGLFVRQLYLTYTNEDVATSVIA   108

1pho_A 093   GRNLGALYDVEAWTD-M-FPEF----G-GDSSAQTDNFM--TKRASGLATY----------------RNTDFFGVIDGLNLTLQYQGK--NENRD-----   159
51dv_A 109   GKQQLNIIWTDNGVDGLVGTGVKVVNNSIDGLTLAAFAVDSFMAAEQGSDLLGQSTYVGNGKNNNDSFKLDSIG--NLYGAAAVGSYDLAGGQFNPQLWL   206

1pho_A 160   -VKKQNGDGFGTSLTYDFG---GSDFAISGAYTNSDRTNEQNLQSRGTGKRAEAWATGLKYDANNIYLATFYSETRKM-----------T------PI--   236
51dv_A 207   AYWDQVAFFYAVDAAYSTTIFDGINWTLEGAYLGNSLDSELD--DKKTYANGNLFALKGSIEVNGWDASLGGLYYGDKEKASTVVIEDQGNLGSLLAGEE   304

1pho_A 237   -T-------GGFANKTQNFEAVAQYQFDFGLRPSLGYVLSKGKDIEG-IGDEDLVNYIDVGATYYFNKNMSAFVDYKINQLDSDNK-LNINNDDIVAVGM   326
51dv_A 305   IFYTTGSRLNGDTGRNIFGYVTGGYTFNETVRVGADFVYGGTKTEATTHLGGGKKLEAVARVDYKYSPKLNFSAFYSYVNLDQGVNTNESADHSTVRLQA   404

1pho_A 327   TYQF     330
51dv_A 405   LYKF     408
```

Int Num 1308
E-value 9.8E-6

```
2por_A 002   VKLSGDARMGVMYNGDDW-----------NFSSRSRVLFTMSGTTDSGLEFGASFKAHESV------------GAETGEDGTVFLSG--AFGKIEMGDAL   076
51dv_A 013   VDVSGVLRYRYDTGNFDKNFLNNSNLNNSKQDHKYRAQVNFSAAIADNFKAFVQFDYNAVDGGTGVDNATNAEKGLFVRQLYLTYTNEDVATSVIAGKQQ   112

2por_A 077   GASEALFG--D-L-YE---VGYTDL---DDRGGNDIPYLTGDE-RLTAEDNP--------VLLYTY-SAGA-FSVAASMSDG--KVG-E------TSEDD   146
51dv_A 113   LNIIWTDNGVDGLVGTGVKVVNNSIDGLTLAAFAVDSFMAAEQGSDLLGQSTYVGNGKNNNDSFKLDSIGNLYGAAAVGSYDLAGGQFNPQLWLAYWDQV   212

2por_A 147   AQEMAVAAAYTFG---N--YTVGLGYEKIDSPDT----ALMADMEQLELAAIAKFGATNVKAYYADGELDRD--------FARA---VF--DL--TP-VA   221
51dv_A 213   AFFYAVDAAYSTTIFDGINWTLEGAYLGNSLDSELDDKKTYANGNLFALKGSIEVNGWDASLGGLYYGDKEKASTVVIEDQGNLGSLLAGEEIFYTTGSR   312

2por_A 222   AAATAVDHKAYGLSVDSTFG-ATTVGGYVQVLDIDT-------IDDVTYYGLGASYDLGGGASIVGGIADNDLP--------NSDMVADLGVKFKF   301
51dv_A 313   LNGDTGRNIFGYVTGGYTFNETVRVGADFVYGGTKTEATTHLGGGKKLEAVARVDYKYSPKLNFSAFYSYVNLDQGVNTNESADHSTVRLQALYKF   408
```

Int Num 1731
E-value 9.5E-6

```
2zfg_A 007   DGNKVDLYGKA-VGLHYFSKGNGENS---YGGNGDMTYARLGFKGETQINSDLTGYGQWEYNFQGNNSE---GADAQTGNKTRLAFAGLKYA-DVGSFDY   098
51dv_A 009   AIKDVDVSGVLRYRYDTGNFDKNFLNNSNLNNSKQDHKYRAQVNFSAAIADNFKAFVQFDYNAVDGGTGVDNATNAEKGLFVRQLYLTYTNEDVATSVIA   108

2zfg_A 099   GRNYGVVYDALG-YT-DMLPEFGGDTA-YSD-----DFF--VGRVGGVAT----------------YRNSNFFGLVDGLNFAVQYLGK--NERDT-----   165
51dv_A 109   GKQQLNIIWTDNGVDGLVGTGVKVVNNSIDGLTLAAFAVDSFMAAEQGSDLLGQSTYVGNGKNNNDSFKLDSIG--NLYGAAAVGSYDLAGGQFNPQLWL   206

2zfg_A 166   -ARRSNGDGVGGSISYEYE-----GFGIVGAYGAADRTNLQEAQPLGNGKKAEQWATGLKYDANNIYLAANYGETRNA-----------TPI--TNKFT-   245
51dv_A 207   AYWDQVAFFYAVDAAYSTTIFDGINWTLEGAYLGNSLDSELDD--KKTYANGNLFALKGSIEVNGWDASLGGLYYGDKEKASTVVIEDQGNLGSLLAGEE   304

2zfg_A 246   ----N---TSGFANKTQDVLLVAQYQFDFGLRPSIAYTKSKAKDVEG-IGDVDLVNYFEVGATYYFNKNMSTYVDYIINQIDSDNK-LGVGSDDTVAVGI   336
51dv_A 305   IFYTTGSRLNGDTGRNIFGYVTGGYTFNETVRVGADFVYGGTKTEATTHLGGGKKLEAVARVDYKYSPKLNFSAFYSYVNLDQGVNTNESADHSTVRLQA   404

2zfg_A 337   VYQF     340
51dv_A 404   LYKF     408
```

Int Num 2194
E-value 3.7E-9

```
3nsg_A 005   NKDGNKLDLYGKAVGRHVWTTTGDSK--------NADQTYAQIGFKGETQINTDLTGFGQWEYRTKADRAE----GEQQNSNLVRLAFAGLKY--AEVGS   090
51dv_A 007   EEAIKDVDVSGVLRYRYDTGNFDKNFLNNSNLNNSKQDHKYRAQVNFSAAIADNFKAFVQFDYNAVDGGTGVDNATNAEKGLFVRQLYLTYTNEDVAT-S   105

3nsg_A 091   IDYGRNYGIVYDVESYTDMAPYFSGET-----WGGA-----YTDNYMTSRAGG------------------LLTYRNSDFFGLVDGLSFGIQYQGKN--   159
51dv_A 106   VIAGKQQLNIIWTDNGVDG-LVGTGVKVVNNSIDGLTLAAFAVDSF-MAAEQGSDLLGQSTYVGNGKNNNDSFKLDSIGNL------YGAAAVGSYDLAG   197

3nsg_A 160   QDNHS-----INSQNGDGVGYTMAYEFD-----GFGVTAAYSNSKRTNDQQDRDGNGDRAESRAVGAKYDANNVYLAAVYAETRNMS--------IVENT   241
51dv_A 198   GQFNPQLWLAYWDQVAFFYAVDAAYSTTIFDGINWTLEGAYLGNSLDSELDD-KKTYANGNLFALKGSIEVNGWDASLGGLYYGDKEKASTVVIEDQGNL   296

3nsg_A 242   ----V------TD---TVEMANKTQNLEVVAQYQFDFGLRPAISYVQSKGKQLNGAGGSADLAKYIQAGATYYFNKNMNVWVDYRFNLLDENDYSSSYVG   328
51dv_A 297   GSLLAGEEIFYTTGSRLNGDTGRNIFGYVTGGYTFNETVRVGADFVYGGTKTEATTHLGGGKKLEAVARVDYKYSPKLNFSAFYSYVNLDQGVNT-NESA   395

3nsg_A 329   TDDQAAVGITYQF     341
51dv_A 396   DHSTVRLQALYKF     408
```

Int Num 2866
E-value 1.1E-8

```
4d65_A 008   GNKLDVYGQIDVRHYFADAKSGE--------DGDDSRVRLGFKGDTQITDQLIGFGRFEWETSTNKA------ETSNDNQNRLAYAGLKFA-DYGSLDYG   092
51dv_A 010   IKDVDVSGVLRYRYDTGNFDKNFLNNSNLNNSKQDHKYRAQVNFSAAIADNFKAFVQFDYNAVDGGTGVDNATNAEKGLFVRQLYLTYTNEDVATSVIAG   109
```

```
4d65_A 093      RNYGVIYDTNAWTDVLPLWGADT-----M-DQ-ED--TF--MMGRNRN-L--L------------TYRNNNGFGYIDG-LSFALQYQGKN--GDQNKSTG      163
51dv_A 110      KQQLNIIWTDNGVDG-LVGTGVKVVNNSIDGLTLAAFAVDSFMAAEQGSDLLGQSTYVGNGKNNNDSFKLDS---IGNLYGAAAVGSYDLAGGQFNPQLW      205

4d65_A 164      SSALDNNGDGYGFSTAYELG----WGLSIGGGYSNSSRTPSQNNIKTGATGKRAEAWNVGSKLELDELYLAAMYGQTLNT-----------TRF---GDD      245
51dv_A 206      LAYWDQVAFFYAVDAAYSTTIFDGINWTLEGAYLGNSLDSELDD---KKTYANGNLFALKGSIEVNGWDASLGGLYYGDKEKASTVVIEDQGNLGSLLAG      302

4d65_A 246      ------D---AEAIANKTENLELVALYSFDFGLTPSIGYNQSKGKNLGN-YGNKDLVKYIAVGASYDFNKNMAAVIDYKINLLKDNQFTDDYGINTDNVL      335
51dv_A 303      EEIFYTTGSRLNGDTGRNIFGYVTGGYTFNETVRVGADFVYGGTKTEATTHLGGGKKLEAVARVDYKYSPKLNFSAFYSYVNLDQGVN--TNESADHSTV      400

4d65_A 336      GLGLIYQF      343
51dv_A 401      RLQALYKF      408
```

Int Num 3213
E-value 4.1E-9

```
5fvn_A 005      NKDGNKLDLYGKVDGLHYFSDDDSQ---------DGDQTYMRLGFKGETQVNDQLTGYGQWEYQIQGNSG-----E-NENNSWTRVAFAGLKF--GDAGS      087
51dv_A 007      EEAIKDVDVSGVLRYRYDTGNFDKNFLNNSNLNNSKQDHKYRAQVNFSAAIADNFKAFVQFDYNAVDGGTGVDNATNAEKGLFVRQLYLTYTNEDVAT-S      105

5fvn_A 088      FDYGRNYGVVYDVTSWTD--VLPEFG---GDTYGS--D-NF---MQQRGN-G--FA------------TYRNSDFFGLVDG-LNFAVQYQGKNGSASGED      160
51dv_A 106      VIAGKQQLNIIWTDNGVDGLVGTGVKVVNNSIDGLTLAAFAVDSFMAAEQGSDLLGQSTYVGNGKNNNDSFKLDSI---GNLYGAAAVGSYDLAGGQFNP      202

5fvn_A 161      QTNNGRTELRQNGDGVGGSITYNLG--E--GFGIGTAVSSSKRTSSQNDLTYGNGDRAETYTGGLKYDANNIYLAAQYTQTYNA-----------TRV--      243
51dv_A 203      QLW--LAYWDQVAFFYAVDAAYSTTIFDGINWTLEGAYLGNSLDSELDD--KKTYANGNLFALKGSIEVNGWDASLGGLYYGDKEKASTVVIEDQGNLGS      298

5fvn_A 244      --G------N-----LGWANKAQNFEVVAQYQFDFGLRPSVAYLQSKGKDLENGYGDQDLLKYVDVGATYYFNKNMSTYVDYKINLLDDKEFTRNAGIST      330
51dv_A 299      LLAGEEIFYTTGSRLNGDTGRNIFGYVTGGYTFNETVRVGADFVYGGTKTEATTHLGGGKKLEAVARVDYKYSPKLNFSAFYSYVNLDQGVNT--NESAD      396

5fvn_A 331      DDIVALGLVYQF      332
51dv_A 397      HSTVRLQALYKF      408
```

Int Num 3231
E-value 8.3E-9

```
5nxr_A 006      KDGNKLDVYGKVDVRHYFASADKGKKS-----EDGDDSRVRLGVKGDTQITDQLTGFGRFEWETKTNKA------ENEGENKNRLAYAGLKFA-DFGSID      093
51dv_A 008      EAIKDVDVSGVLRYRYDTGNFDKNFLNNSNLNNSKQDHKYRAQVNFSAAIADNFKAFVQFDYNAVDGGTGVDNATNAEKGLFVRQLYLTYTNEDVATSVI      107

5nxr_A 094      YGRNYGVVYDTNAWTD--VFPLW---GADTMA------QTDNFMTSRNRN-L--L------------TYRNNNAFGYVDG-LSFALQYQGKNGDNNKSSA      166
51dv_A 108      AGKQQLNIIWTDNGVDGLVGTGVKVVNNSIDGLTLAAFAVDS-FMAAEQGSDLLGQSTYVGNGKNNNDSFKLDS---IGNLYGAAAVGSYDLAGGQFNPQ      203

5nxr_A 167      ---GMAKDNGDGYGFSTAYELGW----GVTLGGGYSSSSRTPNQKAGVVTSEGDSYYSATGKRAQAWNVGGKFDANNVYLAAMYGQTQNT----------      249
51dv_A 204      LWLAYWDQVAFFYAVDAAYSTTIFDGINWTLEGAYLGNSLDSELDD-----------KKTYANGNLFALKGSIEVNGWDASLGGLYYGDKEKASTVVIED      292

5nxr_A 250      -SRY----GD----------LDLIANKTENVELVAQYLFDFGLKPSIGYNQSKGKNLGNGYDNQDLVKYISVGSYYYFNKNMSAVVDYKINLLKDNDFTK      334
51dv_A 293      QGNLGSLLAGEEIFYTTGSRLNGDTGRNIFGYVTGGYTFNETVRVGADFVYGGTKTEATTHLGGGKKLEAVARVDYKYSPKLNFSAFYSYVNLDQGVNT-      391

5nxr_A 335      EYGINTDNVLGLGLVYQF      352
51dv_A 392      -NESADHSTVRLQALYKF      408
```

Loop to hairpin

Int Num 372
E-value 4.6E-4

```
1prn_A 161      TEEFGIAADWSND-----MISLAAAYTTDAGG------IVDNDIAFVGAAYKFNDAGTVGLNWYDNGLSTAGDQVTLYGNYAF--GAT-TVRAYV-SDID      245
3sy7_A 210      YRQYYLNSNYTIPLASDQSLGFDFNIYRTNDEGKAKAGDISNTTWSLAAAYTLDAH-TFTLAYQKVHGDQ-PFDYIGFGRNGSGAGGDSIFLANSVQYSD      307

1prn_A 246      R-AGADTAYGIGADYQFA----EGVKVSGSVQSG      274
3sy7_A 308      FNGPGEKSWQARYDLNLASYGVPGLTFMVRYING      341
```

Int Num 380
E-value 6.7E-4

```
1prn_A 190      DNDIAFVGAAYKFNDAGTVGLNWYDNGLSTAGDQVTLYGNYAFG--ATTV-R-AYVSDIDRAGADTAYGIGADYQFA----EGVKVSGSVQSG      274
3szd_A 248      DNRTVSALFSARYGLH-TLYLGLQKVSGDD---G----WMRVNGTSGGTLANDSYNASYD-NPGERSWQLRYDFDFVGLGLPGLTFMTRYLHG      331
```

Int Num 385
E-value 6.7E-4

```
1prn_A 160      VTEEFGIAADWSNDMISLAAAYTTDAGGIVDNDIAFVGAAYKFNDAGTVGL-NWYDNGLSTAGDQVTLYGNYAFG----A-TTVRAYVSDIDR-------      247
3t0s_A 244      DNRNLNAMLTLRAGAHAFGIGVQKMIGN----DAFPVLNGYT-TPY-VANLMAYQTFTR-PQEKSWQLRYDYDFAGLGLPGLNLMTRYVQGRDIDRGAGR      336

1prn_A 248      -AGADTAYGIGADYQFA----EGVKVSGSVQSG      274
3t0s_A 337      ADDSEWERNTDLSYVIQSGPLKSVALKWRNITY      369
```

Int Num 395
E-value 1.0E-3

```
1prn_A 190      DNDIAFVGAAYKFNDAGTVGLNWYDNGLSTAGDQVTLYGNYAFGA-TTVRAYVSDIDR-AGADTAYGIGADYQFA----EGVKVSGSVQSG      264
4frx_A 280      DNRAFSGLFTYTVSGH-SIGAGYQILNGDSDF-PFLNR-GDGEGSTAYLITDVQIGKFQRAGERTWQVRYGYDFATVGVPGLTFNTIYLSG      367
```

```
Int Num 910
E-value 2.8E-4
2fgr_A  168    VGRYIGGYVAYDNGPLSASLGLAQQKTAVGGLATDRDEITLGASYNFGVAKLSGLLQQTKFKRDIGGDIKTNSYMLGASAPVG----GVGEVKLQYALYD    267
4frx_A  280    DNRAFSGLFTYTVSGHSIGAGYQILNGDSD-----FPFLNRGDG-EGSTAYLITDVQIGKF-----QRAGERTWQVRYGYDFATVGVPGLTFNTIYLSGD    370

2fgr_A  266    QKA---IDSKAHQITLGYVHNLS----KRTALYGNLAFLKNKD    299
4frx_A  369    KIKTARGDQSEWERDISLAYVIPDGTFKGLGFTWKNASFRSGL    279
```

```
Int Num 922
E-value 6.8E-4
2fgr_A  168    VGRYIGGYVAYDNGPLSASLGLAQQKTAVGGLATDRDEITLGASYNFGVAKLS-GLLQQTKFKRDIGGDIKTNSYMLGASAPVG-----GVGEVKLQYAL    261
5dl5_A  274    TNNIWAISGTYATGPHSVMLAYQQNTGNVG-----Y-DYGQ-NA--DGFQSIYLPNSYMSDF-----IGNHEKSAQIQYNVDFGKLGVLPGLNWTTAFVY    359

2fgr_A  262    YDQ-KA----IDSKAHQITLGYVHNLS----KRTALYGNLAFLKNKD    299
5dl5_A  360    GWDIKVRNVTDDAQEREFFNQVKYTVQSGFAKDASLRIRNSYYRASD    273
```

```
Int Num 2791
E-value 2.8E-5
4aui_A  017    AMADVTLYGTIKAGVETSRSVAHHGAQADRVKTATEIADLGSKIGFKGQEDLGNGLKAIWQLEQKAYVSG--------TDTGWGNRQSFIGLKG--GFGK    106
5ldv_A  009    AIKDVDVSGVLRYRYDTGNFDKNFL-NNSNLNNSK--QDHKYRAQVNFSAAIADNFKAFVQFDYNAVDGGTGVDNATNAEKGLFVRQLYLTYTNEDVATS    105

4aui_A  107    VRVGRLNN--ILKDT----GNFNP-------WEGKSYY-SGLSNIAQ--PEE---------------------R-HVS----VRYDSP-EFAGFSGSVQ    162
5ldv_A  106    VIAGKQQLNIIWTDNGVDGLVGTGVKVVNNSIDGLTLAAFAVDSFMAAEQGSDLLGQSTYVGNGKNNNDSFKLDSIGNLYGAAAVGSYDLAGGQFNPQLW    205

4aui_A  163    YVPNDNSGKNRSESYHAGFNYKND---GFFVQYAGSYKRHNYT--------TEKHQVHRLVGGYDHDALYASVAVQQQDAKL--------T-WRD----D    238
5ldv_A  206    LAYWD----QVAFFYAVDAAYSTTIFDGINWTLEGAYLGNSLDSELDDKKTYANGNLFALKGSIEVNGWDASLGGLYYGDKEKASTVVIEDQGNLGSLLA    301

4aui_A  239    N-------------SHNSQTEVATTVAYRFG-NVTPRVSYAHGFKGSVYD-ADHDNTYDQVVVGAEYDFSKRTSALVSAGWLQKGKGAE---KFVATVGG    320
5ldv_A  302    GEEIFYTTGSRLNGDTGRNIFGYVTGGYTFNETVRVGADFVYGGTKTEATTHLGGGKKLEAVARVDYKYSPKLNFSAFYSYVNLDQGVNTNESADHSTVR    401

4aui_A  321    VGLRHKF     327
5ldv_A  402    LQALYKF     408
```

```
Int Num 2965
E-value 1.2E-4
6ehb_A  181    GEDGYSLSAIYTFGDTGFNVGAGYADQDDQNEYMLAASYRMEN-LYFAGLFTDGELAKDVDYTGYELAAGYKLG----Q-AAFTATYNNAETAK-----K    269
4fso_A  258    DNRALSLLLAYAQG--GHTLSAGWQRMNGASSM----PYLDGSNPYLANYLQVNDF-ANPEERSWQLRYDFDLRSVGVPGLSFMTRYVNGDHIRLANGDE    350

6ehb_A  270    TSADNFAIDATYYFK----PNFRSYISYQFNLLDSDKASKVASEDELAIGLRYDF     320
4fso_A  351    GKEWERDIELKYIVQSGRFKDLSLRLRNATYRTDFER--SARDVDEVRLIASYNL     403
```

```
Int Num 2983
E-value 1.9E-4
6ehb_A  181    GEDGYSLSAIYTF-GDTGFNVGAGYADQDDQNEYMLAASYRME--NLYFAGLFTDGELAKDVDYTGYELAAGYKLGQ-----AAFTATYNNAETAK----    268
4fsp_A  262    DNRSFSGSLTYRLRN--GQAFGLGYQRMSGDHGF----PYLEGTDPYLVNF-GQYNDF-AEAGESSWQLRYDCDFAPLGVPGLSLMTRYFSGHGAKPKGA    353

6ehb_A  269    -KTSADNFAIDATYYFK----PNFRSYISYQFNLLDSDKASKVASEDELAIGLRYDF     320
4fsp_A  354    DGSREWERDSDLRYVLQGGALKGLGLVWRNATYRSAF-----SRDIDENRLYLTYEL     405
```

```
Int Num 3159
E-value 5.1E-4
6ehb_A  181    GEDGYSLSAIYTFGDTGFNVGAGYADQDDQNEYMLAASYRMENLY-F-AGLFTDGELAKDVDYTGYELAAGYKLG---Q-AAFTATYNNAETA-------    245
5dl6_A  187    DNDLYHAHFELKHQ--NHKFIFGTFQHHGDTAF----PYLTGGETGLLIDTWP-GEF-LNPKEKAYSFRYEYDFKEYVPGLCFMTRYTTGHNIYAPNLGG    305

6ehb_A  246    KKTSADNFAIDATYYFK----PNFRSYISYQFNLLDSDKASKVASEDELAIGLRYDF     298
5dl6_A  306    TNLKERETDFDLGYTVQSGWLKNLGLRARYAIYDNNMLSTANIKPVNETRINIDYTW     362
```

```
Int Num 3235
E-value 7.0E-9
6ehd_A  005    KSDAGTVDFYGQLRTELKFLEDKDP---------TIGSGSSRAGVDANYTVNDSLALQGKVEFALKDS-----------GDMYVRNHILGVKT--NFGKF    082
5ldv_A  007    EEAIKDVDVSGVLRYRYDTGNFDKNFLNNSNLNNSKQDHKYRAQVNFSAAIADNFKAFVQFDYNAVDGGTGVDNATNAEKGLFVRQLYLTYTNEDVATSV    106

6ehd_A  083    SFGKQWTT--SDDV--Y-G-AD---YSY-FFGGTGLR--Y-G---TLSDA----------------------LHDSQ----VKYVY--EADSFWVKAGY    137
5ldv_A  107    IAGKQQLNIIWTDNGVDGLVGTGVKVVNNSIDGLTLAAFAVDSFMAAEQGSDLLGQSTYVGNGKNNNDSFKLDSIGNLYGAAAVGSYDLAGGQFNPQLWL    206

6ehd_A  138    GFPEDNAKQELAELYVGATF--G-DLAVHAGGGQNRDKAFKVGSNTVGTTTTDIKADVTNSYFEVTGEYTIGDALIGVTYYNAELD-----------V--    221
5ldv_A  207    AYWDQ--VAFFYAVDAAYSTTIFDGINWTLEGAYLGNSLDSELD--------DKKTYANGNLFALKGSIEVNGWDASLGGLYYGDKEKASTVVIEDQGNL    296

6ehd_A  222    ----EN-----------NPLVIDEDAISVAGTYKVADKTKLYAGYEYVMQEANT---GADEDGTLVYLGVEYKFASWARVYAEYGYGDGTTLGYTNKGSD    303
5ldv_A  297    GSLLAGEEIFYTTGSRLNGDTGRNIFGYVTGGYTFNETVRVGADFVYGGTKTEATTHLGGGKKLEAVARVDYKYSPKLNFSAFYSYVNLDQGVNT-----    391

6ehd_A  304    AEVKATKVDSANNFGIGARYYW     325
5ldv_A  392    -----NESADHSTVRLQALYKF     408
```

Loop to hairpin with alternate alignments

```
Int Num 378 – Loop to hairpin
E-value 1.9E-4
1prn_A 190      DNDIAFVGAAYKFNDAGTVGLNWYDNGLSTAGDQVTLYGNYAFGAT--TVRAYVSDIDR-AGADTAYGIGADYQFA----EGVKVSGSVQSG      274
3sys_A 245      DSHTVYGLFSAGIGLH-TFYLGLQKVGGDS-------GWQSVYGSSGRSMGNDMFNGNFTNADERSWQVRYDYDFVGLGWPGLIGMVRYGHG      328

Int Num 379 – Alternate alignment, also loop to hairpin
E-value 2.8E-4
1prn_A 160      VTEEFGIAADWSNDMISLAAAYTTDAGGIVDNDIAFVGAAYKFNDAGTVGLNWYDNGLS-TAGDQVTLYGNYAFG----A-TTVRAYVSDIDR-------      246
3sys_A 245      DSHTVYGLFSAGIGLHTFYLGLQKVGGDS--GWQSVYG-SS---GR-SMGNDMFNGNFTNADERSWQVRYDYDFVGLGWPGLIGMVRYGHGSNATTKAGS      337

1prn_A 247      AGADTAYGIGADYQFA----EGVKVSGSVQSG      274
3sys_A 338      GGKEWERDVELGYTVQSGPLARLNVRLNHASN      369

Int Num 1628 - Loop to hairpin
E-value 9.1E-5
6ehb_A 181      GEDGYSLSAIYTFGDTGFNVGAGYADQDDQNEYMLAASYRME--NLYFAGLFTDGELAKDVDYTGYELAAGYKLG----Q-AAFTATYNNAETAK-----      268
2y0l_A 256      DNKSLNGMFTYSLG--NHAFGAAWQRMNGDDAF----PYLEGSNPYLVNFV-QVNDF-AGPKERSWQLRYDYDFVGLGIPGLTFMTRYVKGDNVELAGQS      347

6ehb_A 269      -KTSADNFAIDATYYFK----PNFRSYISYQFNLLDSDKASKVASEDELAIGLRYDF      320
2y0l_A 348      GEGREWERNTELQYVFQSGALKNLGIRWRNATFRSNF-----TRDIDENRLIVSYTL      399

Int Num 1627 – alternate alignment
E-value 1.0E-5
6ehb_A 182      EDGYSLSAIYTFGDTGFNVGAGYADQDD-QNEYMLAASYRME----NLYFAGLFTDGELAK-----DVDYTGYELAAGYKLGQAAFTATYNNAETAK---      268
2y0l_A 189      DHFDLGGLDYKLTD-QLTASYHYSNLQDVYRQHFVGLLHSWPIGPGELTSDLRFARSTDSGSAKAGGIDNKSLNGMFTYSLGNHAFGAAWQRMNGDDAFP      287

6ehb_A 269      ------------------KTSADNFAIDATYYFK----PNFRSYISYQFNLLD      299
2y0l_A 288      YLEGSNPYLVNFVQVNDFAGPKERSWQLRYDYDFVGLGIPGLTFMTRYVKGDNV      341

Int Num 2550 – loop to hairpin
E-value 1.5E-4
6ehb_A 181      GEDGYSLSAIYTFGDTGFNVGAGYADQDDQNEYMLAASYRME--NLYFAGLFTDGELAKDVDYTGYELAAGYKLG----Q-AAFTATYNNAETAK-----      268
3szv_A 256      DNRAFGALFSLRLG--AHAVAAGYQRISGDDPYP----YIAGSDPYLVNF-IQIGDF-GNVDERSWQLRYDYDFGALGLPGLSFMSRYVSGDNVARGAAN      347

6ehb_A 269      KTSADNFAIDATYYFK----PNFRSYISYQFNLLDSDKASKVASEDELAIGLRYDF      320
3szv_A 348      DGKEWERNTDLGYVVQSGPLKNLGVKWRNATVRSNF-----ANDLDENRLILSYSL      398

Int Num 2551 – alternate alignment
E-value 2.8E-5
6ehb_A 181      GEDGYSLSAIYTFGDTGFNVGAGYADQDD-QNEYMLAASYRME-----NLYFAGLFTDGELAK---DVDYTGYELAAGYKLGQAAFTATYNNAETAK---      268
3szv_A 189      SDAFDFAGGDYRLTP-ALTASLHQGRLKDIYRQTFAGLVHTLDLGGQRSLKSDLRFARASEDGGFRELDNRAFGALFSLRLGAHAVAAGYQRISGDDPYP      287

6ehb_A 269      ------------------KTSADNFAIDATYYFK----PNFRSYISYQFNLLDS      300
3szv_A 288      YIAGSDPYLVNFIQIGDFGNVDERSWQLRYDYDFGALGLPGLSFMSRYVSGDNVA      342

Int Num 2948 – Loop to hairpin
E-value 1.0E-3
6ehb_A 180      NGEDGYSLSAIYTFGDTGFNVGAGYADQDDQNEY-MLAASYRM-ENLYFAGLFTDGELAKDVDYTGYELAAGYKLGQ-----AAFTATYNNAETAK----      268
4frx_A 279      VDNRAFSGLFTYTVS--GHSIGAGYQILNGDSDFPFLNRGDGEGSTAYLITDVQIGKF-QRAGERTWQVRYGYDFATVGVPGLTFNTIYLSGDKIKTARG      375

6ehb_A 269      KTSADNFAIDATYYFK----PNFRSYISYQFNLLDSDKA---SKVASEDELAIGLRYDF      320
4frx_A 376      DQSEWERDISLAYVIPDGTFKGLGFTWKNASFRSGLPAAGSSNNQRDQDENRLIVSYTL      434

Int Num 2947 – alternate alignment
E-value 4.1E-4
6ehb_A 181      GEDGYSLSAIYTFGDTGFNVGAGYADQDD-QNEYMLAASYRM--E--NLYFAGLFTDGELA-------------------------KDVDYTGYELAAGY      250
4frx_A 192      SNKFYYAGGDYKVNK-DLTLQYYYGNLDDFYKQHFLGLIHNWQIGPGVLKTDLRAFDSSSDGKNGSRSGRADGYVSSGYYGSGVTKGEVDNRAFSGLFTY      290

6ehb_A 251      KLGQAAFTATYNNAETAK--------------------------KTSADNFAIDATYYFK----PNFRSYISYQFNLLDS      300
4frx_A 291      TVSGHSIGAGYQILNGDSDFPFLNRGDGEGSTAYLITDVQIGKFQRAGERTWQVRYGYDFATVGVPGLTFNTIYLSGDKIK      371

Int Num 3173 – Loop to hairpin
E-value 1.5E-4
6ehb_A 181      GEDGYSLSAIYTFGDTGFNVGAGYADQDDQNEYMLAASYRME---NLYFAGLFTDGELAKDVDYTGYELAAGYKLGQ----------AAFTATYNNAETA      267
5dl7_A 263      DNRHISGLFGLNYQ--NHTVSLGYMQSFGSTGL----PFLSGTESPVVLD--FMSSDY-SNKDEKVYSIRYEYDFKNARIGDVSLNGLRFMTRYAKGEDI      353
```

```
6ehb_A  268       K-------KTSADNFAIDATYYFK----PNFRSYISYQFNLLDSDKASKVASEDELAIGLRYDF      320
5dl7_A  354       DLLQYGDQRFKEDSLEFDLGYKIPEGKLKGLGMRARFSHYRNDMPTNMTFHSANETRLNVDYTF      417
```

Int Num 3174 - alternate alignment
E-value 7.4E-5

```
6ehb_A  181       GEDGYSLSAIYTFGDTGFNVGAGYADQDD-QNEYMLAASYRME-----NLYFAGLFTDGELAK-----DVDYTGYELAAGYKLGQAAFTATYNNAETAK-          268
5dl7_A  195       TDSFYTLGGSYQLK--DYRLRAYHAELKDIYQQQFLGFNGKQPLNDQLNFLSDVRFFNSEETGSKKIGEVDNRHISGLFGLNYQNHTVSLGYMQSFGSTG          292

6ehb_A  269       ---------------------KTSADNFAIDATYYFKP---------NFRSYISYQFNLLDS      300
5dl7_A  293       LPFLSGTESPVVLDFMSSDYSNKDEKVYSIRYEYDFKNARIGDVSLNGLRFMTRYAKGEDID      354
```
